# Spontaneous Collapse of the Walls of the Antrum

**Published:** 1852-04

**Authors:** 


### ARTICLE XI.

### *Spontaneous Collapse of the Walls of the Antrum.*

A paper by White Cooper was read, (and illustrated by a portrait and cast,) of a case of collapse of the antrum. The patient was a respectable young Irish woman, of healthy constitution, and strong frame, who, nine years ago, perceived a dusky mark beneath the left eye; after a time this extended down by the side of the nose, and was followed by a sinking of the cheek in that situation; there was, however, neither pain nor uneasiness. After this had existed nearly seven years, gradually increasing in extent, she applied to Mr. Cooper on account of the tear flowing over the cheek. Palliative measures were adopted, and this unpleasant symptom subsided. This was early in 1849, and since that time the sinking of the anterior wall of the antrum has steadily continued to increase, and has now given rise to considerable deformity, the appearance closely resembling that which would have arisen had a large portion of the superior maxillary bone been removed, and the integument sunk. The teeth on the affected side were in a most unhealthy state, and two were removed in 1849, by Mr. Alfred Canton, in the hope that the morbid action might be arrested; but such has not been the case. Mr. Cooper has not been able to find a similar case related in any work.

After the reading of the case, various opinions in respect to its nature were offered. There was no syphilitic taint, and no external injury. It was conjectured that the deformity might be natural, that it was not very uncommon for the left side of the face to be smaller than the right; that it might have arisen from mollities ossium; that the antrum might have fallen in, from the contraction consequent upon the spontaneous cure of a disease in that cavity, by which the anterior wall was dragged inwards; that it might have arisen from falling in of the anterior wall from obstruction of the opening of the antrum into the nasal duct, and consequent atmospheric pressure. None of these solutions, however, were regarded by the author as conclusive or satisfactory.—*London Lancet.*

